# Distinct representations of configural and part information across multiple face-selective regions of the human brain

**DOI:** 10.3389/fpsyg.2015.01710

**Published:** 2015-11-06

**Authors:** Golijeh Golarai, Dara G. Ghahremani, Jennifer L. Eberhardt, John D. E. Gabrieli

**Affiliations:** ^1^Department of Psychology, Stanford UniversityStanford, CA, USA; ^2^Department of Psychiatry and Biobehavioral Sciences, University of California, Los AngelesLos Angeles, CA, USA; ^3^Harvard-MIT Division of Health Sciences and Technology (HST) and Department of Brain and Cognitive Sciences, Massachusetts Institute of TechnologyBoston, MA, USA

**Keywords:** occipito-temporal cortex, FFA, amygdala, STS, face, configuration, parts, holistic representation

## Abstract

Several regions of the human brain respond more strongly to faces than to other visual stimuli, such as regions in the amygdala (AMG), superior temporal sulcus (STS), and the fusiform face area (FFA). It is unclear if these brain regions are similar in representing the configuration or natural appearance of face parts. We used functional magnetic resonance imaging of healthy adults who viewed natural or schematic faces with internal parts that were either normally configured or randomly rearranged. Response amplitudes were reduced in the AMG and STS when subjects viewed stimuli whose configuration of parts were digitally rearranged, suggesting that these regions represent the 1st order configuration of face parts. In contrast, response amplitudes in the FFA showed little modulation whether face parts were rearranged or if the natural face parts were replaced with lines. Instead, FFA responses were reduced only when both configural and part information were reduced, revealing an interaction between these factors, suggesting distinct representation of 1st order face configuration and parts in the AMG and STS vs. the FFA.

## Introduction

Human faces convey socially relevant information about emotion, intention and identity. Coordinated activity across a network of human brain regions underlies face processing, where by core regions in this network are thought to be specialized in processing specific aspects of facial information (Haxby et al., 2002; Said et al., 2011). For example, the amygdala (AMG) responds to faces, especially to facial expressions of fear (Adolphs and Spezio, 2006). Face selective regions along the superior temporal sulcus (STS) are involved in detecting facial movements associated with eye gaze, speech, and expression of emotions and intentions (Puce et al., 1998; Allison et al., 2000; Thompson et al., 2007; Cohen Kadosh et al., 2010; Esterman and Yantis, 2010). And face-selective regions along the fusiform gyrus (FG), collectively known as the fusiform face area (FFA) are implicated in face detection and identity recognition (Kanwisher et al., 1998; Golby et al., 2001; Grill-Spector et al., 2004; Kanwisher and Yovel, 2006). Much research on the face-processing network has focused on elucidating the distinct functional properties of each region, the interactions among these regions, and their common pathways. However, it remains unknown what specific facial cues differentially engage these brain regions in face processing.

Faces share a common set of parts (eyes, nose, etc.) arranged in a typical spatial configuration within the boundaries of the face (also known as the 1st order configuration: nose above the mouth, eyes above the nose), but vary in the appearance of the parts and the fine grain spatial relations among those parts. Numerous behavioral experiments have shown that both configural and part information in faces contribute to accurate face processing. For example, disruption of the 1st order face configuration by inversion of face stimuli or rearrangement of facial features reduced subjects' performance during tasks involving emotion recognition (McKelvie, 1995; Collishaw and Hole, 2000; Prkachin, 2003; Lobmaier and Mast, 2007; Derntl et al., 2009; Schwaninger et al., 2009) and led to substantial decrements in performance during identity recognition tasks (Tanaka and Farah, 1993). Indeed, there is evidence that FFA responses to faces are based on the whole face (Rossion et al., 2000) and sensitive to subtle changes in the spatial relations among face parts (Rhodes et al., 2009). Thus, one hypothesis suggests that processing of the 1st order configural information in faces may be a common step during performance of various face-related tasks. Moreover, given that the 1st order configuration is a key characteristic among all natural faces, disruption of this information may lead to substantial signal decrements across several face-selective regions, such as the AMG, STS, and FFA. However, other studies suggest that the degree of reliance on configural and part information in faces varies depending on the task and brain region. For example, subjects correctly guessed the expressed emotion in single features, e.g., happiness in a smiling mouth (Leppänen et al., 2007), or direction of gaze in an eye. Likewise, viewing the white of the eyes in fearful vs. neutral faces was sufficient to evoke AMG responses (Whalen et al., 2004). Thus, single facial features might be sufficient for accurate processing of expressive faces via the AMG or STS (Puce et al., 1998; Adolphs et al., 2005). In contrast, performance during identity recognition undergoes a substantial decrement when healthy adults relied on facial features (Tanaka and Farah, 1993; Schiltz and Rossion, 2006). Moreover, the FFA but not the STS showed sensitivity to subtle changes in the spatial relations among facial features (Rhodes et al., 2009). Indeed, poor face recognition performance in patients with acquired prosopagnosia following injury to the ventral stream is associated with feature-by-feature processing of faces (Busigny and Rossion, 2010; Van Belle et al., 2011; Busigny et al., 2014). Together these findings suggest an alternative hypothesis, namely that configural and part information in faces are differentially represented across brain regions involved in processing of expressive facial signals (e.g., AMG and STS) vs. regions involved in processing of face identity (e.g., FFA). Specifically, the AMG and STS may be more sensitive to the appearance of face parts, whereas the FFA may be relatively more sensitive to configural information. Such differential representations of configural and part information across face-selective regions would suggest the contribution of non-overlapping and perhaps local neural circuits in processing these types of facial information in each region.

Moreover, configural and part information may interact within each region. Indeed, in the macaque infero-temoral (IT) cortex, neural responses to facial features depend on their spatial position within the boundaries of the whole face (Freiwald et al., 2009), suggesting an interaction between part and configural information among face-selective neurons in the IT cortex. However, the relative contribution of configural and part information or the potential interactions among these factors within face-selective regions of AMG, STS, and FFA in humans is not clear.

In humans, electrophysiological studies have shown that disruption of the natural configuration of face parts by arbitrary rearrangement of internal parts within the frame of the face images altered the amplitude and timing of face-specific, temporal cortex responses (i.e., N170) to normal vs. rearranged face stimuli (Bentin et al., 1996; Rossion et al., 1999; Eimer, 2000; Halgren et al., 2000; Sagiv and Bentin, 2001; Liu et al., 2010). However, the regional localization of this signal modulation is not clear, as fMRI studies of configural and part processing have provided conflicting results. For example, early studies found no effect in the response amplitudes of face selective regions along the FG when the overall face configuration was disrupted (Grill-Spector et al., 1998; Kanwisher et al., 1998; Haxby et al., 1999; Lerner et al., 2001; Joseph et al., 2006; Collins et al., 2012), although more recent studies provide evidence of signal reductions in the FG (Collins et al., 2012) or the FFA (Liu et al., 2010). Specifically, Collins et al. showed signal reduction in response to face stimuli after disruption of 1st order face configuration within the anatomical boundaries of the FG, but no signal modulations were found in the AMG or STS, consistent with the greater sensitivity to configural information within sub-regions of the FG, relative to AMG or STS (Collins et al., 2012). However, it is not clear from this study if the sensitivity to configural information within the anatomical boundaries of the FG, overlap with the face-selective regions of FFA. Another study reported substantial reductions in the FFA responses to rearrangement of face parts while responses in the STS remained unchanged, also suggesting a unique sensitivity of the FFA to the 1st order configuration of face parts in contrast to a lack of sensitivity in the STS (Liu et al., 2010). However, in this study response amplitudes of STS to images of natural faces were low and thus the lack of sensitivity in STS to the 1st order configuration may have been the result of low signal to noise ratio in this region. Thus, the relative sensitivity of the AMG, STS, and FFA to the normal configuration of face parts remains unclear.

A related question is whether or not face selective regions of the AMG, STS, and FFA are similar in representing the natural appearance of face parts. Separate studies have shown that all of these regions represent face parts, especially the eye region (Puce et al., 1998, 2003; Allison et al., 2000; Morris et al., 2002; Wheaton et al., 2004; van Belle et al., 2010; Issa and DiCarlo, 2012). However, the relative sensitivity of face-selective regions to the natural appearance of face parts or the potential interaction of configural and featural representations among the AMG, STS, and FFA remains to be determined.

Here we asked if face-selective regions in the AMG, STS, and FFA are equally sensitive to the 1st order configuration and appearance of face parts. We performed fMRI in two experiments while participants viewed images of natural faces, or face images that were digitally transformed to remove the 1st order face configuration by rearrangement of internal face parts (rearranged faces, in Experiments 1 and 2) or to remove the natural appearance of face parts by replacement of natural parts with simple lines (schematic faces, Experiment 2), or both manipulations. We expected that brain regions which represent the overall face configuration would respond more strongly to naturally configured faces than to rearranged faces, and that regions representing the natural appearance of face parts would respond more strongly to faces with natural parts than to schematic faces.

## Methods

### Participants

Twenty healthy European American adults (8 females) ages 18–35 participated in Experiment 1. Two participants were removed from further analysis due to excessive motion during fMRI (see below). Eight (4 females) of the 18 also participated in Experiment 2. All participants were right handed with normal or corrected vision and without any past or current neurological or psychiatric conditions, or structural brain abnormalities. Informed consent was obtained according to the requirements of the Panel on Human Participants in Medical Research at Stanford University.

### Stimuli and pilot behavioral test

**Figure 1:**
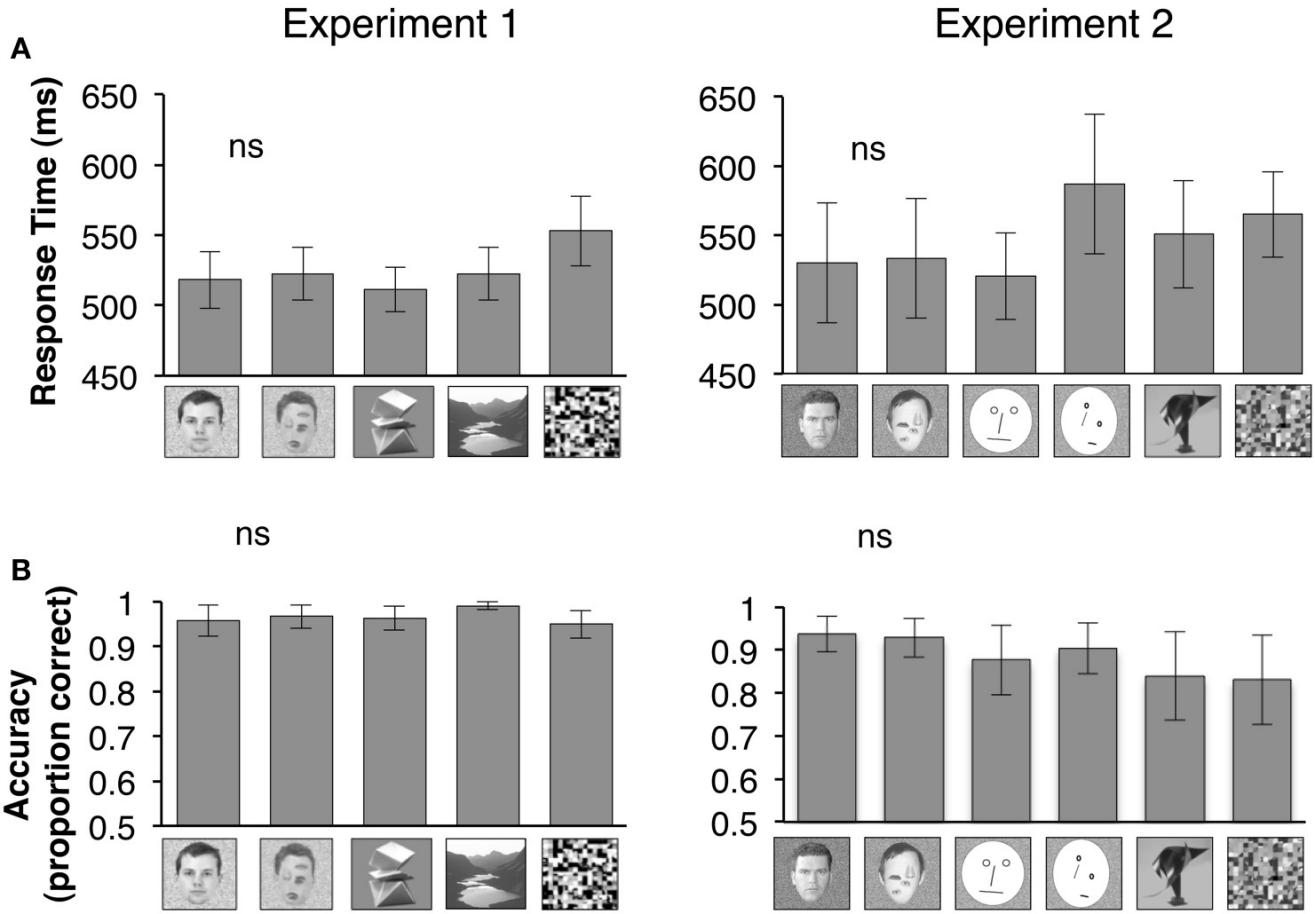
**Bar graphs show mean of all participants' median response times during a 1-back task that subjects performed during fMRI**. An example of each stimulus type is shown below the corresponding bar graph. **(A)** Participants' response times during the 1-back task is plotted for Experiment 1 (*n* = 18) or Experiment 2 (*n* = 7) for each category of visual stimuli displayed below the bar graphs. *ns:* Response times to natural vs. rearranged faces were not statistically different. **(B)** Participants' accuracy in performing the 1-back during Experiment 1 or Experiment 2 is plotted based on proportion correct (maximum = 1). *ns:* Accuracy in performance of 1-back task for natural vs. rearranged faces were not statistically different.

In Experiment 1, stimuli included 60 gray-scale photographic images for each of the following five categories: natural faces, rearranged faces (digitally rendered by moving the internal face parts to random positions within the normal hairline using Adobe Photoshop), novel objects (abstract sculptures), indoor and outdoor scenes, and textures (scrambled versions of the other categories; Figure 1A). In Experiment 2, participants viewed another set of natural and rearranged natural faces, and novel objects as in Experiment 1, as well as 60 schematic faces and 60 rearranged schematic faces (Figure 1B). Visual stimuli were not repeated between Experiments 1 and 2. All natural and rearranged-natural faces were of European American males, standardized to show a frontal view of the face above the neck, displaying neutral expressions with no eyeglasses or jewelry, and were placed against a uniform gray background.

Schematic faces consisted of two eyes, a nose and a mouth within the face outline. These face parts were represented by simple lines and ovals (blurred using a Gaussian function in Adobe Photoshop), which did not resemble faces or face parts when presented in rearranged configurations. This was confirmed by a pilot study where 10 participants (not involved in fMRI) saw five samples of the rearranged schematics followed by five correctly configured schematic faces and were asked to identify each picture presented one at a time in response to the question: “What is this?” Rearranged schematics were labeled as faces or face parts in 4 of 50 trials, and correctly configured schematic faces were labeled as faces in 50 of 50 trials. These results demonstrate that the schematic stimuli were perceived as faces only when configured as a face (i.e., they were perceived as faces purely on the basis of the configuration of the internal parts, and the parts alone were not interpreted as either a face or parts of a face).

### FMRI and behavioral task

During fMRI, each image category was presented during five pseudo randomly ordered blocks. Blocks were 14 s long followed by 14 s of fixation background. Stimulus images were presented at 1 s intervals, each for 970 ms, followed by a 30 ms fixation baseline. Each image was presented only once, except for two randomly places images in each block, which were presented twice successively for a one-back task. Thus, there were two instances of the 1-back task during each block and these were randomly located within each block. Participants were instructed to look at each image and press a button using their right index finger whenever they detected identical images that appeared successively (i.e., a 1-back task). Responses during the 1-back task were collected in 20/20 subjects in Experiment 1 and 7/8 subjects in Experiment 2.

Images were projected onto a mirror mounted on the MRI coil (visual angle ~ 14°). Images were presented and responses were recorded via a Macintosh G3 computer using Matlab and the PsychToolbox extensions Psychtoolbox (www.psychtoolbox.org). Average response times for each stimulus category were calculated as the group mean of subjects' median time for correct responses during the one-back task.

### Scanning

Brain imaging was performed on a 3 Tesla whole-body General Electric Signa MRI scanner (General Electric, Milwaukee, WI) with a quadrature birdcage head coil. Participants used a bite bar (made of dental impression material) to stabilize the head position and reduce motion-related artifacts during the scans. First, a high-resolution 3D Fast SPGR anatomical scan (124 sagittal slices, 0.938 × 0.938 mm, 1.5 mm slice thickness, 256 × 256 image matrix) of the whole brain was obtained. Next, a T2-weighted fast spin echo in-plane with a slice prescription identical to that of the functional scan was acquired. Functional images were obtained using a T2^*^-sensitive gradient echo spiral-in/out pulse sequence using blood oxygenation level-dependent (BOLD) contrast (Glover and Law, 2001). Full brain volumes were imaged using 22 slices (4 mm thick plus 1 mm skip) oriented parallel to the line connecting the anterior and posterior commissures. Brain volume images were acquired continuously with a repetition time (TR) of 1400 ms, TE = 30 ms, flip angle = 70°, field of view = 240 mm, 3.75 × 3.75 mm in-plane resolution, 64 × 64 image matrix. Data for Experiments 1 and 2 were acquired during separate runs in the same session, each run was approximately 14 min.

### Data analysis

**Figure 2:**
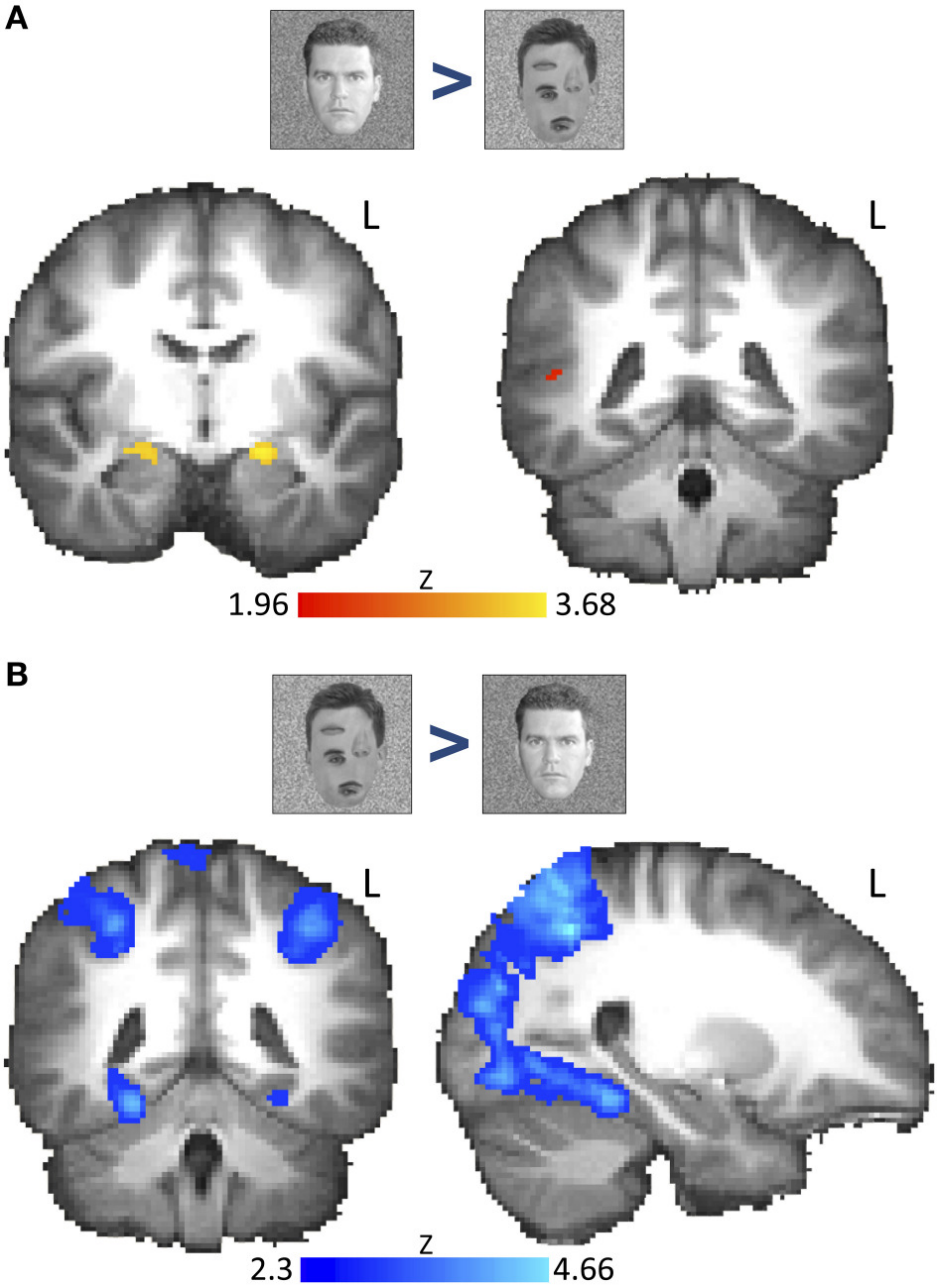
**Voxel-wise group fMRI results for the contrast “natural faces > rearranged natural faces” and the reverse contrast**. Brain images show thresholded z-statistic maps from group analysis (*N* = 18) overlaid on a group-averaged high-resolution T1 anatomical image. **(A)** The contrast “*natural faces* > *rearranged natural faces”* revealed bilateral AMG activation (family-wise error correction using bilateral AMG, STS, and ventral-occipito-temporal cortex as *a priori*-determined search space, *P* < 0.05; left image: AMG: MNI: *Y* = 8). Right STS activation was found only at uncorrected thresholds (right image: STS: MNI: *Y* = −42, height threshold: *P* < 0.01). **(B)** The contrast “*rearranged natural faces* > *natural faces”* showed activation along the medial FG and parietal regions (see Table S1). Whole-brain activations were cluster-corrected (cluster-threshold: *P* = 0.05, height threshold: *Z* > 2.3). L, left hemisphere. Color bar indicates z-statistic range.

**Figure 3:**
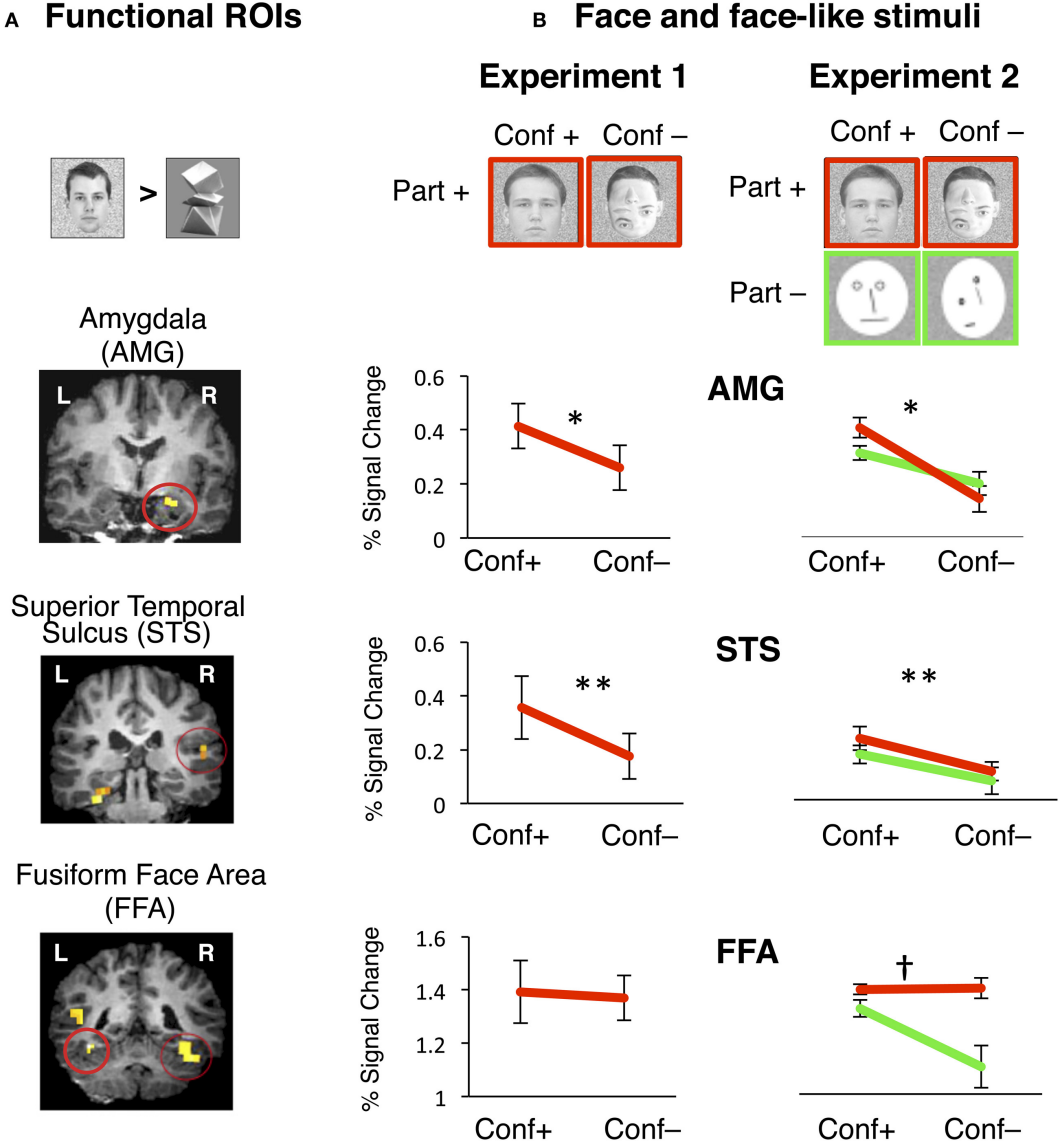
**Face selective functional ROIs were defined using data from one experiment and signals were extracted during an independent experiment**. **(A)** Face selective ROIs in the AMG, posterior STS and FFA were defined by the contrast of *natural faces* > *novel objects, P* < 0.001. Examples of individual t-maps with this contrast are overlaid on coronal slices of high-resolution T1 volume from a representative participant. Functional ROIs are high-lighted by a red circle. **(B)** During Experiment 1, visual stimuli included natural faces with the normal configuration of face parts (“conf +”) and face-like stimuli where internal parts were randomly rearranged within the face boundary (“conf –”). During Experiment 2 visual stimuli included face-like images that retained the natural appearance of face parts (images with red outline, “parts +”) or to face-like schematics (green out line, “parts –”). Each type of stimulus was presented either by retaining the 1st order configuration of internal face parts (“conf +”), or random rearrangement of internal parts (“conf –”). Independent analysis of response amplitudes during Experiment 1 to face-like stimuli in the right hemisphere from face-selective regions of AMG, STS, and FFA. Red lines show response amplitudes to face stimuli that retained the 1st order configural information (“conf +”) and stimuli with internal parts randomly rearranged (“conf –”). Error bars show ± SEM. **Right AMG:** Removal of configural information significantly reduced response amplitudes in the right AMG. ^*^conf: *p* = 0.03, *n* = 7. **Right STS:** Removal of configural information significantly reduced response amplitudes in the right STS. ^**^conf: *p* = 0.0001, *n* = 6. **Right FFA:** Removal of configural information did not reduce response amplitudes in the right FFA in the presence of part information. *n* = 8. Independent analysis of response amplitudes during Experiment 2 to face-like stimuli in the right hemisphere from face-selective regions of AMG, STS, and FFA. Red lines show response amplitudes to face stimuli that retained natural part information (“part +”). Green lines show response amplitudes to schematic faces (“part –”). Responses to face stimuli are plotted for the subtypes that retained the 1st order configural information (“conf +”) and stimuli with internal parts randomly rearranged (“conf –”). Error bars show ± SEM. **Right AMG:** Removal of configural information significantly reduced response amplitudes in the right AMG in the presence (red line) or absence (green line) of part information. ^*^conf: *p* = 0.01, *n* = 7. **Right STS:** Removal of configural information significantly reduced response amplitudes in the right STS in the presence (red line) or absence (green line) of part information. ^**^conf: *p* = 0.0001, *n* = 6. **Right FFA:** Removal of configural information did not reduce response amplitudes in the right FFA in the presence of part information (red line), but did so in the absence of part information (green line), revealing a significant interaction between factors of part and configural information. †**conf X part:** *p* = 0.007, *n* = 8.

**Figure 4:**
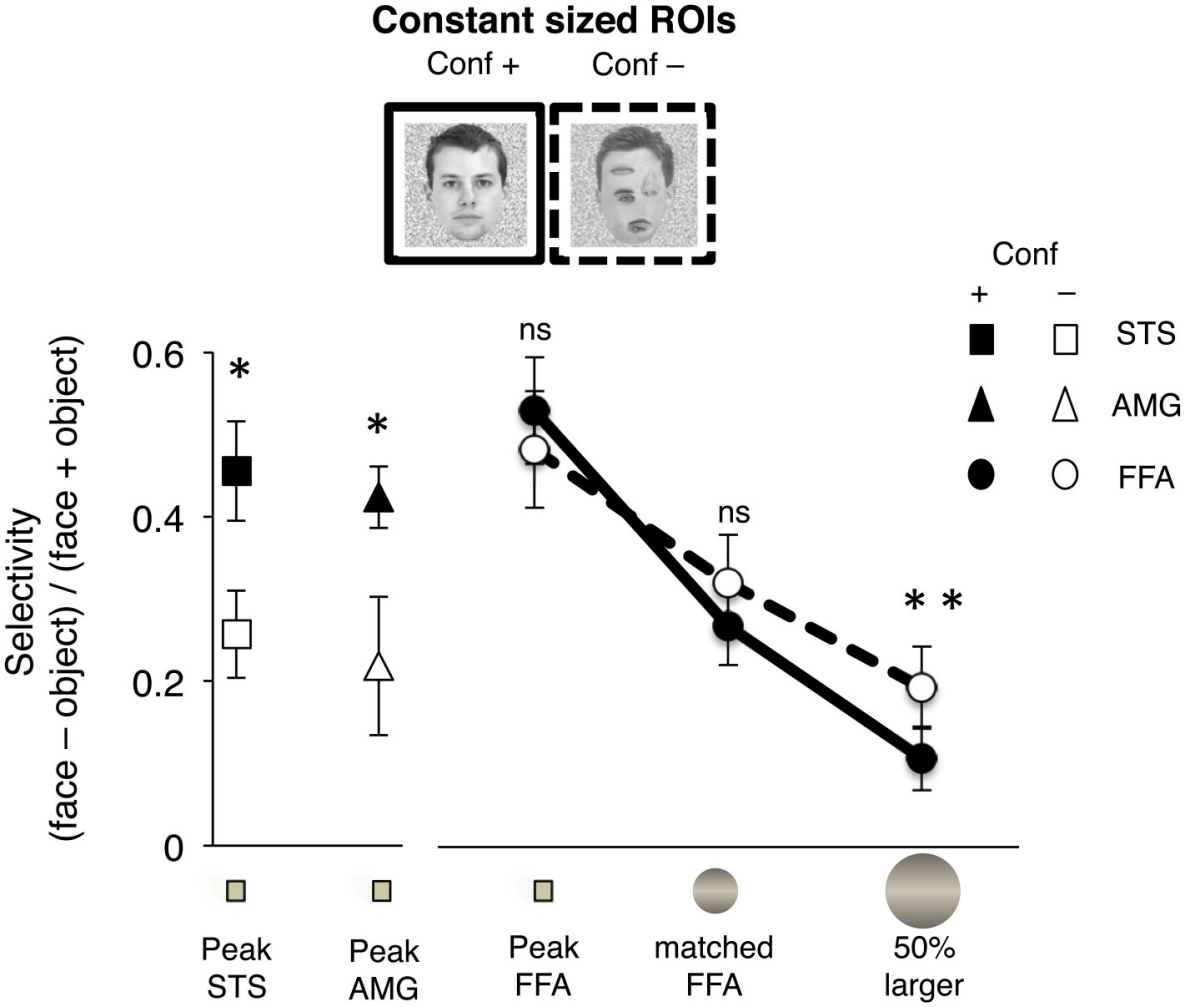
**Measure of selectivity for natural (“Conf +”) or rearranged face (“Conf –”) stimuli is plotted for three adjacent voxels including the peak of the AMG, STS, and FFA, and two additional concentric ROIs in the FG**. Selectivity was calculated based on the difference of % signal change for each type of face stimulus vs. objects (*[face – object]/[face* + *object]*). The ROIs were defined for each subject as three adjacent voxels including the peak selectivity for faces (“*peak”*), a concentric sphere matched in volume to the size of the average FFA across all subjects (“*matched FFA”*) and a sphere that was 50% larger in volume (“*50% larger”*). Response amplitudes to natural faces were significantly higher in the peak voxels of the STS and AMG (^*^P < 0.001, *n* = 18). There were no significant differences in selectivity for natural vs. rearranged faces at the FFA peak or the sphere matched to FFA size (*ns, p* > 0.25). Selectivity was significantly higher for rearranged than natural faces in the “50% larger” ROI (^**^P = 0.048, *n* = 18).

Data were analyzed using the FSL (5.0.8) toolbox from the Oxford Centre for fMRI of the Brain (www.fmrib.ox.ac.uk/fsl) for group analysis (Figure 2) and Statistical Parametric Map (SPM) software package (SPM2, Wellcome Department of Cognitive Neurology) for region of interest (ROI) analyses (Figures 3, 4, Figures S1–S3). The first 10 functional volumes were discarded to allow for T1 equilibration. Functional scans were motion corrected (Jenkinson et al., 2002). As noted above, data from two participants were not used for further analysis due to excessive motion (>2 mm), leaving 18 subjects in Experiments 1 and 8 subjects who also participated in Experiment 2.

### Voxel-wise analysis

Voxel-wise fMRI analyses were performed using the FSL (5.0.8) toolbox from the Oxford Centre for fMRI of the Brain (www.fmrib.ox.ac.uk/fsl). After motion correction, all non-brain matter was removed using FSL's brain extraction tool. Data were spatially smoothed using a 5 mm full-width-half maximum Gaussian kernel. Registration was conducted through a three-step procedure, whereby BOLD images were first registered to the inplane structural image, then to the SPGR high resolution T1 structural image, and finally into standard [Montreal Neurological Institute (MNI)] space (MNI avg152 template), using 12-parameter affine transformations (Jenkinson and Smith, 2001). Registration from SPGR structural images to standard space was further refined using FNIRT nonlinear registration (Andersson et al., 2007a,b). Statistical analyses at the single-subject level were performed in native space, with the statistical maps normalized to standard space prior to higher-level analysis.

Whole-brain statistical analysis was performed using a multi-stage approach to implement a mixed-effects model treating participants as a random effects variable. Regressors of interest were created by convolving a *delta* function representing block onset times with a canonical (double-*gamma*) hemodynamic response function. Six motion parameters were included as covariates of no interest to account for variance associated with residual motion. Two additional metrics of motion were also included as covariates: frame-wise displacement and a combination of the temporal derivative of the time series and root mean squared variance over all voxels (Power et al., 2014). For all analyses, time-series statistical analysis was carried out using FILM (FMRIB's Improved Linear Model) with local autocorrelation correction (Woolrich et al., 2001) after high-pass temporal filtering (Gaussian-weighted LSF straight line fitting, with sigma = 33 s).

For this group-level analysis, the FMRIB Local Analysis of Mixed Effects (FLAME1) module in FSL was used (Beckmann et al., 2003; Woolrich et al., 2004), and a one-sample *t*-test was performed at each voxel for each contrast of interest. Z (Gaussianised T) statistic images were thresholded using cluster-corrected statistics with a height threshold of *Z* > 2.3 (unless otherwise noted) and a cluster probability threshold of *p* < 0.05, corrected using the theory of Gaussian Random Fields (Worsley et al., 1992), either at whole-brain or within specified masks containing regions of interest. All data were subjected to robust outlier deweighting (Woolrich, 2008). For the contrast *natural faces* > *rearranged natural faces* (Figure 2A), we restricted analyse to regions relevant for face processing, including bilateral ventral occipito-temporal cortex, STS, and AMG. A mask consisting of these regions, anatomically defined via the Harvard-Oxford Probabilistic Atlas, was applied to the contrast images prior to group-level statistical inference. We also examined the reverse contrast, *rearranged faces* > *natural faces*, without restricting to this mask, using a more exploratory approach (Figure 1B, also see Table S1).

Anatomical loci of all activations were verified using a sectional anatomy atlas (Duvernoy and Bourgouin, 1999).

### Functional region of interest (ROI) analyses

#### Independent analyses

We conducted independent analyses of percent signal change within functionally defined ROIs and generated two separate data sets: (i) defined functional ROIs using Experiment 2 and extracted signals from Experiment 1 (Figure 3C, Figure S1); (ii) defined functional ROIs using Experiment 1 and extracted signals from Experiment 2 (Figure 3D, Figure S3). None of the stimuli were repeated between Experiments 1 and 2. Both experiments included blocks of natural and rearranged natural faces, but only Experiment 2 included blocks of schematic and rearranged schematic faces.

To define face-selective regions, we used spatially smoothed (6 mm FWHM) functional images in each subject's native space and the contrast of *natural faces* > *novel objects* (at *p* < 0.001 uncorrected, cluster size > 3 voxels), and selected supra-threshold voxels within the anatomical boundaries of the AMG, the posterior superior temporal gyrus (STS) or the FG. These latter activations were centered in the FG and extended medially to the collateral sulcus. When more than one cluster of face-activation was evident along the FG, we selected the more extensive activation.

#### Constant size, peak, and spherical ROIs

In each subject we selected three neighboring voxels at the peak of face-selectivity based on the highest *T*-value for the contrast (*natural faces* > *novel objects*) in the AMG, STS, and lateral FG in Experiment 1. We also defined two additional concentric spherical ROIs in the lateral FG, one matched to the size of the average FFA volume across all subjects, and another matched to 150% of the average FFA volume. We extracted the percent signal change to face like stimuli and objects during Experiment 1 from all voxels within these ROIs. Then we calculated the relative selectivity for face like stimuli as (*[f* − *o]/[f* + *o]*), where “*f*” is the percent signal change to natural or rearranged faces, and “*o*” is the percent signal change to novel objects (see Figure 4). Thus, these ROIs were all centered at the peak of individually defined face-selective regions, but the specific selection of voxels that were included in this analysis were not functionally defined and independent of the signals that we extracted from these voxels.

#### Dependent analyses

For Experiment 1, in one analysis we functionally defined ROIs using Experiment 1 data and extracted signals from the same experiment (see Figure S2).

**Percent BOLD signal change** for each stimulus category was determined by extracting the raw time-course data for each ROI. For each subject, if a given anatomical location showed <3 supra-threshold voxels for the contrast of interest, that ROI was not included in the analysis. Data were then band-pass filtered (high-pass = 0.0052 Hz cut-off; low-pass = SPM's synthetic hemodynamic response function, Gaussian temporal filter at 4 s FWHM cut-off), shifted in time by 6 s to account for the hemodynamic lag, averaged within each stimulus block (14 s), and then across blocks of each category. Individual time series data were converted to percent signal change relative to the mean activation during fixation blocks, and normalized to the mean activation during texture blocks.

#### Statistical analysis

Differences in the percent signal change between stimulus categories (repeated measure) and ROIs were evaluated using repeated measures analysis of variance (rmANOVA) or paired *t*-tests. All reported statistics are based on two-tailed tests, unless otherwise noted.

## Results

### Behavioral results

During fMRI participants (*n* = 18) performed a one-back task while viewing image categories that were presented in blocks. In Experiment 1, these categories included images of natural faces, natural face images after digital rearrangement of internal parts (rearranged faces), novel objects (abstract sculptures), scenes (outdoor, indoor, buildings), and scrambled images of the other categories (Figure 1). In Experiment 2, eight subjects (who also participated in Experiment 1) viewed different images of the same categories as in Experiment 1, in addition to simple line drawing of faces (schematic faces: ovals and lines within a large oval outline, arranged in the 1st configuration of face parts) and schematic faces with internal parts randomly rearranged (Figure 1).

Repeated measures analysis of variance (rmANOVA) on response latencies (repeated measures) during the one-back task across visual stimuli showed a significant effect of visual stimulus category when we included all visual stimuli in Experiment 1, but not when we limited the comparison to face stimuli in a *post-hoc* analysis in Experiment 1 [**all stimuli**: *F*_(4, 17)_ = 3.10, *P* = 0.03; **face stimuli**: *F*_(1, 17)_ = 0.25, *P* = 0.63]. There were no category effects in the response latencies during Experiment 2 [**all stimuli**: *F*_(5, 6)_ = 1.51, *P* = 0.25; **face stimuli**: *F*_(3, 6)_ = 0.24, *P* = 0.65, Figure 1A, due to technical issues we did not record the 1-back responses in 1 subject during Experiment 2].

Accuracy in performing the one-back task was high (82–99%) across all stimulus categories, and did not differ significantly when we examined responses to all visual stimuli or only face stimuli in Experiment 1 [**all stimuli**: *F*_(4, 17)_ = 1.41, *P* = 0.24; **face stimuli**: *F*_(1, 17)_ = 0.30, *P* = 0.60] or in Experiment 2 [**all stimuli**: *F*_(5, 6)_ = 2.52, *P* = 0.18; **face stimuli**: *F*_(3, 6)_ = 1.85, *P* = 0.19, *rmANOVA*, Figure 1B]. These findings suggest that participants paid equal attention to all stimuli during fMRI.

### Imaging results

#### Differential fMRI responses to natural and rearranged faces: voxel-wise group analysis

To determine regions across the brain that respond to the 1st order configural information in faces, we examined the contrast of *natural faces* > *rearranged natural faces*. After correcting for multiple comparisons (restricting to AMG, FG, and STS), we found bilateral AMG activation (Figure 2A). However, clusters within bilateral STS were only found using uncorrected thresholds (*P* < 0.01).

To our surprise, we found no activation in the FG or the wider ventral or occipital temporal cortex whether in the corrected or uncorrected group analyses (Table S1A). This lack of activation was not due to non-specific BOLD artifacts as we found a robust activation to the reverse contrast (*rearranged natural faces* > *natural faces*, cluster-corrected, *P* < 0.05, *Z* > 2.3) along the bilateral FG (Figure 2B) as well as several other cortical regions (Table S1A), mostly across occipito-parietal cortex, including the collateral sulcus, parietal and frontal cortices and the precuneus.

These findings suggest that responses in the AMG and possibly STS, but not the FFA are sensitive to disruption of 1st order configural information in faces, supporting a differential representation of 1st order face configuration across these ROIs. Alternatively, the weak STS activation and lack of FFA activation may be due to greater between-subject variability in the location and spatial extent of face-selective regions along the length of the STS and FG respectively. We tested these possibilities by evaluating the response profiles of individually defined functional ROIs below.

#### Independent analyses of percent BOLD signal change during experiment 1 among individually defined functional ROIs

To test the hypothesis that 1st order face configuration is differentially represented across the face-selective regions of AMG, STS, and FFA, we examined the response properties of these regions in an independent analysis. We used an independent experiment (Experiment 2, *n* = 8) as a localizer to functionally define these regions of interest (ROIs) in each subject's native brain space, based on the contrast of (*natural faces* > *novel objects, p* < *0.001*, see Methods). Next, we measured these regions' response amplitudes during Experiment 1 (Figure 3B).

As expected in all three regions of AMG, STS, and FFA, response amplitudes to natural faces were higher than to objects (Figure S1). However, these ROIs varied in their sensitivity to the 1st order configural information in natural faces (Figure 3B). A two-way rmANOVA of response amplitudes to natural vs. rearranged face stimuli across the three face-selective ROIs in the right hemisphere showed significant main effects of ROI and face-type, and a significant ROI by face-type interaction in the right hemisphere [**right: ROI**: *F*_(2, 18)_ = 50.47*, P* < 0.0001, **face type:** *F*_(1, 18)_ = 48.84, *P* = 0.0001, **ROI X face-type**: *F*_(2, 18)_ = 6.1, *P* = 0.009, Figure 3B]. In the left hemisphere, we also found a significant main effect of ROI, indicating variations among ROI responses, but there were no significant effects of face-type [**left: ROI**: *F*_(2, 19)_ = 4.9, *P* < 0.02, **face type:** *F*_(1, 19)_ = 2.25, *P* = 0.14, **face-type X ROI**: *F*_(2, 19)_ = 1.83, *P* = 0.19, *rmANOVA*, Figure S1].

In a series of *post-hoc* analyses on the responses of each ROI, we found that rearrangement of face parts resulted in a significant reduction in response amplitudes in the right, but not in the left AMG [**right:** *t*_(6)_ = 2.64, *p* = 0.034, **left:** *t*_(7)_ = 0.97, *p* = 0.36, *paired t-test*]. Likewise, there was a significant reduction in response amplitudes in the STS bilaterally [**right:** *t*_(5)_ = 7.73, *p* = 0.0001**, left**: *t*_(6)_ = 2.6, *p* = 0.03, *paired t-test*]. This effect was highly consistent in the right hemisphere of all subjects and evident in every right AMG and STS ROI that we tested. In contrast, removal of configural information did not change responses in the FFA in either hemisphere (*t* < 1, *p* > 0.3, *paired t-test*). Together, these data support the hypothesis that the AMG, STS, and FFA differentially represent the 1st order configuration of faces.

#### Percent BOLD signal change among individually defined ROIs during experiment 1 in a dependent analysis

We replicated these results in a dependent analysis of AMG, STS, and FFA responses during Experiment 1 (defined functional ROIs and extracted signals from the same data, *n* = 18, Figure S2). Thus, the lack of modulation to 1st order configural information in the FFA was not a result of variability in FFA localization between experimental runs.

#### Percent BOLD signal change among individually defined ROIs of constant size during experiment 1

Next, we tested the possibility that sensitivity to 1st order face configuration in the FFA may be evident among its more face-selective voxels, and similar to the responses of the AMG and STS. Thus, we selected three adjacent voxels including the peak of face-selectivity in each of the anatomical regions of the AMG, STS, and FFA, and extracted response amplitudes to face and face-like stimuli during Experiment 1 (*n* = 18, Figure 4). We found that response amplitudes to natural faces were significantly higher than responses to rearranged faces around the peak of selectivity in the AMG and STS (*p* < 0.001, *paired t-tests*), but not the FFA (*p* = 0.23).

To examine further the lack sensitivity of FFA responses to rearrangement of face parts, we considered the converse possibility that voxels with lower selectivity for faces within the FFA may show a greater range of responses and more modulation to rearrangement of face parts. Thus, in each subject's FG we also defined two larger concentric spherical ROIs centered at the peak of face selectivity in FG, one matched in volume to the average size of FFA across all subjects and the other matched in volume to 50% larger than the average FFA size. We found no significant difference in the selectivity to images of natural faces vs. rearranged face stimuli in the sphere overlapping the FFA in either hemisphere (*p* > 0.3, *paired t-test*, Figure 4).

Interestingly, there was a trend toward *higher* selectivity for rearranged faces in the larger sphere that extended outside the right FFA (**right:** *p* = 0.05; **left:** *p* > 0.09, *n* = 18, *paired t-test*, Figure 4), consistent with the extended activation along the FG to the contrast of [*rearranged face* > *natural face*] in the group analysis (Figure 2B).

Note that the selection of voxels was based on constant sized ROIs (three voxels in case of the peak ROIs, and based on the group averaged size of the FFA for the concentric spheres), providing an independent analysis of regional response profiles.

Together these findings indicate that in contrast to the responses of STS and AMG, neither the highly face-selective voxels at the peak of the FFA nor the FFA voxels surrounding the peak showed any signal modulation to removal of 1st order face configuration.

#### Independent analyses of percent BOLD signal change during experiment 2 among individually defined functional ROIs

Next, we tested the possibility that FFA's potential sensitivity to removal of 1st order configural information may be masked by its high amplitude responses to the natural appearance of face parts. Thus, in Experiment 2 we manipulated the appearance of face parts and used schematic faces with internal parts that consisted of simple lines, arranged either in the normal face configuration or randomly rearranged within the boundaries of an oval. Then, we examined the responses of the face-selective ROIs (AMG, STS, and FFA) to four types of face-like stimuli: (i) natural faces, (ii) rearranged-natural faces, (iii) schematic faces with the normal face configuration or (iv) rearranged schematic faces (Figure 3B, also see Methods).

Among the face-selective region of AMG, a two-way rmANOVA with factors of configuration and part information on the repeated measures of response amplitudes to face and face-like stimuli showed a significant main effect of configural information and a significant interaction between configural and part information; however, the effect of part information did not reach significance [**right: configural information:** *F*_(1, 6)_ = 71.62, *P* < 0.0001, **part information:** *F*_(1, 6)_ = 0.20, *P* < 0.67, **configural X part information**: *F*_(1, 6)_ = 16.99, *P* < 0.007, Figure 3B]. This interaction was due to a trend toward higher amplitude of responses to natural than to schematic faces only when the natural face configuration was preserved [*t*_(6)_ = 1.9, *p* = 0.04, *one-tailed paired t-test*]. In the left AMG signal amplitudes to faces were close to baseline and differences between face-like stimuli did not reach significance (Figure S3). Thus, the right AMG responses were more sensitive to the presence of the 1st order face configuration than to the appearance of those parts.

Among face-selective regions of STS, a two-way rmANOVA with factors of configuration and part information on the repeated measures of response amplitudes to face and face-like stimuli showed a significant effect of configural information [**right STS: configural information:** *F*_(1, 5)_ = 9.81, *P* = 0.01, *two-way rmANOVA*, Figure 3B], as rearrangement of internal face parts reduced STS responses [**right STS**: *t*_(5)_ > 2.62, *p* < 0.05, *paired t-test*] regardless of the natural or schematic appearance of face parts. However, there were no effects of part information and no interactions between configural and part information [*F*_(1, 5)_ < 1.45, *P* > 0.26, *two-way ANOVA*]. In the left STS there were similar trends toward an effect of configuration as well as a trend toward an effect of part information (*P* = 0.1, *n* = 6, *two-way rmANOVA*, Figure S3). These data confirm that face-selective regions in the right STS are sensitive to the configuration of internal face parts, but less sensitive to the natural appearance of those parts, analogous to AMG responses.

Distinct from the AMG and STS, a similar two-way rmANOVA on responses in the FFA revealed significant main effects of configuration, part information and an interaction between these factors [**right FFA: configural information:** *F*_(1, 7)_ = 20.13, *P* = 0.001, **part information:** *F*_(1, 7)_ = 4.10, *P* = 0.05, **configural X part information**: *F*_(1, 7)_ = 10.36, *P* = 0.007, *rmANOVA*, Figure 3B]. These effects were due to a significant reduction in the response amplitudes to rearranged schematic faces (i.e., removal of both configural and part information) relative to other face-like stimuli, which preserved either or both type of information [*t*_(7)_ > 3.71, *p* < 0.01, *paired t-test*]. Results were similar in the left FFA (Figure S3). Thus, FFA responses were generally unchanged to rearrangement of internal face parts in naturalistic face stimuli or after removal of the natural appearance of face parts in simple schematics, if these retained the 1st order configuration of internal parts. However, simultaneous rearrangement of internal parts and replacement of the parts with simple lines lead to a substantial signal reduction in the FFA (Figure 3B), rendering these responses indistinguishable from FFA response amplitudes to objects (see Figure S3C).

## Discussion

We used fMRI to examine brain responses while participants viewed images of natural faces and images of face-like stimuli that were digitally transformed by rearrangement of internal face parts, replacement of natural parts with lines, or both manipulations. We found evidence for different sensitivities to the 1st order face configuration and the natural appearance of face parts across the three face-selective regions of the AMG, STS, and FFA. Specifically, AMG and STS responses were primarily modulated by the presence of the 1st order configuration of internal face parts, and less so by the natural appearance of those parts. In contrast, FFA responses showed surprisingly little modulation by removal of either the 1st order face configuration or the natural appearance of those parts. Instead, FFA responses were substantially diminished when both types of information were removed. These findings reveal differential representations of configural and part information across face-selective regions of the AMG and STS vs. FFA, suggesting distinct neural mechanisms of configural and part processing among these regions.

Several of our findings support the above interpretations of the data. First, participants' performance on the 1-back task during fMRI showed that accuracy and response times were similar for all face and face-like stimuli, indicating that there were no substantial differences in global attention to these stimuli. Second, four different fMRI analyses converged on the same main findings: (i) Voxel-wise group analyses of fMRI signals in Experiment 1 (Figure 2) revealed that regions in the AMG represent the 1st order configural information in natural faces, as the AMG responded more to natural than to rearranged faces. A similar, but statistically weaker, activation was also evident in the STS. In contrast, no regions in the FG showed this sensitivity. (ii) Independent analyses of ROI responses—by functionally defining ROIs in one experiment and extracting signals from another experiment within each subject's native brain anatomy—confirmed that the AMG and STS differ from the FFA in representing configural and part information (Figure 3). Furthermore, this analysis revealed a unique interaction among these representations, specifically in the FFA. These regional variations in representation of 1st order configural information were consistent in our results from both iterations of independent analyses across the two experiments (using Experiment 1 as localizer and extracting signals from Experiment 2 and vice-versa). (iii) Also consistent were results from analysis of peak responses (in 3 adjacent voxels including the peak) in the AMG, STS, and FFA, and also spherical ROIs in the FFA, which were individually defined, fixed in size and centered at the peak of selectivity in each region and subject (Figure 4). The selection of two voxels adjacent to the peak (and the spherical ROIs in the FFA) was agnostic to the functional properties of these voxels. However, this analysis in the FFA showed no evidence of reduction in response to rearranged vs. natural faces. Importantly, the lack of response modulation to removal of the 1st order configuration in natural faces even in the vicinity of the peak of the FFA ruled out the possibility that this lack of sensitivity in the FFA is due to signal averaging at its boundary, with regions outside of the FFA. (iv) Finally, a dependent analysis of FFA responses during Experiment 1 (Figure S2) confirmed the lack of FFA modulation by 1st order configural information, ruling out the possibility of confounds related to between run variability in localization of FFA. Note that the latter two analyses on data from Experiment 1 had the advantage of higher statistical power due to larger number of subjects (compared to the independent analyses). Yet, these analyses consistently showed signal modulation to removal of 1st order configural information in the AMG and STS and a lack of this modulation in the FFA, even among its peak face-selective voxels. Together, these findings reveal that AMG and STS are sensitive to both configural and part information, but a distinct response profile was found in the FFA responses, suggesting diverging neural pathways for configural and part processing across these regions during viewing of neutral faces.

### Face selective regions of AMG and STS represent the typical face configuration

The sensitivity of face-selective regions in the AMG and STS to the 1st order configuration of faces may be understood in terms of these regions' functional specialization in extracting specific types of facial information, which are depleted in the rearranged face-like stimuli, namely socially relevant facial information. For example, the AMG is involved in recognition of facial affect, and responds to emotionally salient stimuli (Adolphs and Spezio, 2006). Similarly the STS is associated with speech, eye gaze, and emotional expression (Puce et al., 1998; Allison et al., 2000; Hoffman and Haxby, 2000; Materna et al., 2008) and more generally biological motion (Puce and Perrett, 2003; Grossman et al., 2010). The STS is also implicated in inferences about intentions, beliefs, and feelings of other persons and more generally social perception (Yang et al., 2015). Thus, greater AMG and STS responses to natural faces than to rearranged faces may reflect participants' extensive prior experience with natural faces in social contexts, and the paucity of socially relevant information that is conveyed by the rearranged or simple schematic faces.

Second, there is evidence that the STS and AMG extract information from specific facial features. For example, AMG responses to facial expressions of fear are critically dependent on the appearance of the eyes (Morris et al., 2002; Rutishauser et al., 2015). Interestingly, the white regions of the eyes are sufficient to activate AMG responses (Whalen et al., 2004). Other studies have reported that the AMG (in contrast to the visual cortex) is specifically responsive to the low spatial frequency information in fearful facial expressions (Vuilleumier et al., 2003; Winston et al., 2004). Indeed, the low spatial-frequencies in faces retain a disproportionate amount of configural information while losing mostly local part information. Consistent with AMG representation of configural information, our data highlight that, during viewing of neutral faces, removing the overall configuration of face parts substantially reduced AMG or STS responses, but removing the natural appearance of face parts did not substantially modulate these signals.

The sensitivity of AMG and STS to configural information that we found during viewing of neutral faces does not contradict the significance of facial features during processing of affective or communicative facial signals. One possibility is that reliance on part information in AMG and STS may be greater during processing of expressive faces (compared to our findings during viewing of neutral faces). Another possibility is that configural information ensures the efficient detection of affective information from the relevant face parts (e.g., from the eyes) during observers' typical patterns of eye movements in scanning the internal features of face stimuli. Future studies of eye-movements during viewing of rearranged faces will be useful in determining the significance of 1st order configuration of internal face parts in automatic targeting of observers' gaze upon face parts during free viewing. Likewise, in our study we used neutral faces to define face-selective voxels in the AMG and STS. However, voxel selection criteria based on expressive faces may yield a different spatial spread across the STS and different functional properties. Thus, future experiments using expressive faces will be important in revealing the relative contributions of configural and part information to AMG and STS responses.

### FFA responses to naturalistic face parts and the typical face configuration

In contrast to the STS and AMG, responses of the FFA were virtually identical when participants viewed natural faces or natural face parts that were randomly rearranged within the face outline, across two experiments and a number of analyses. This lack of modulation was not due to low sensitivity in our measurements, given that the reverse contrast revealed response modulation to these stimuli in nearby regions in the FG (Figure 2B). Indeed, our findings are consistent with a number of earlier fMRI studies that found small or no differences in FFA activations when face configuration was manipulated by inversion (Kanwisher et al., 1998; Beauchamp et al., 1999; Joseph et al., 2006), randomly fragmenting face images by up to 16 divisions (Grill-Spector et al., 1998; Lerner et al., 2001), or rearrangement of face parts (Collins et al., 2012). However, in these studies face inversion, fragmentation or rearrangement preserved some information on the spatial relations among the internal face parts, leaving open the possibility that the spatial relations among these parts may be critical in evoking FFA responses. Our results rule out this possibility.

A more recent study by Liu et al. found evidence for signal reductions in response to rearranged faces in the FFA but not the STS (Liu et al., 2010), in apparent contrast to our findings. However, this reduction was reported for a combination of rearranged faces with natural face parts and cartoon like face parts (i.e., internal face parts that were replaced with dark ovals, Figure 3 in Liu et al.). This signal reduction to the combined removal of face configuration and part information is in fact consistent with the reduced FFA responses to rearranged cartoon faces in our data. Based on our data, we hypothesize that the rearranged cartoon like faces, which lacked both the 1st order configuration and natural appearance of face parts, primarily drove Liu et al.'s reported findings. In turn, our data suggest a more complex scenario, and provide evidence for an interaction between 1st order configuration and part information in the FFA.

Our results pose an apparent paradox. Namely, behavioral studies have shown that rearrangement of natural faces slow face detection (Homa et al., 1976; van Santen and Jonides, 1978; Purcell and Stewart, 1988; Rolls et al., 1994) and hamper face recognition (Tanaka and Farah, 1993). Also, small variations in the shape and configuration of face parts across individual identities are readily detected during face recognition and identity discrimination, and failure to detect these small variations are associated with reduced face recognition performance (Le Grand et al., 2003). Furthermore, there is evidence for whole face processing in the right FFA (Rossion et al., 2000) and signal modulation in the FFA in response to subtle variations in the spatial relations among face parts (Rhodes et al., 2009). These data would suggest that the normal face configuration is critical for operation of the neural systems that are involved in face detection and recognition, such as the FFA (Golby et al., 2001; Ishai et al., 2002; Grill-Spector et al., 2004; Winston et al., 2004; Kanwisher and Yovel, 2006) and would specifically predict response reductions in the FFA for rearranged faces, contrary to our findings. Another hypothesis suggests that responsiveness to faces in the FFA depends on the extensive experience that most individuals have with natural faces (Gauthier et al., 2000; McGugin et al., 2014). This notion of “expertise” would also predict reductions in FFA responsiveness to rearranged faces, a category of visual stimuli with which participants had no previous experience. Our results counter these convergent predictions, showing that novel configurations of internal face parts were just as effective in activating the FFA, as were natural faces.

Why were FFA responses reduced by the rearrangement of schematic but not natural face parts? One possibility is that the variability and salience of rearranged natural faces leads to higher activations among face-selective regions, compensating for any signal reduction due to loss of configural information. Indeed, the higher variability in the configuration of internal face parts might reduce the potential for adaptation effects in the FFA. Although our results were consistent when we examined FFA voxels at peak selectivity for faces, or voxels that included a wider range of selectivity across the FFA (Figure 4), we cannot rule out FFA's signal reduction due to adaptation to the 1st order configuration in natural and schematic faces in our data. Also, the bizarre appearance of the rearranged faces might increase their salience and face-selective regions' response amplitudes to these faces, compared to natural faces. In case of the FFA, these effects might be sufficient to compensate for any signal reduction due to loss of the 1st order configuration. Testing these possibilities requires a systematic analysis of image similarity and adaptation responses in the FFA across the various face-like stimuli in future studies. However, the contrast between the unchanging response profiles of the FFA to these face-like stimuli compared to the AMG and STS, both of which showed substantial signal reduction to rearranged faces, indicate that the relative contribution of these opposing factors vary across these face selective regions. These findings support the notion that configural and part information are processed along neural pathways that are distinct for FFA vs. AMG or STS.

A second possibility is that FFA responds to incomplete facial information in an all-or-none manner, perhaps involving pattern-completion mechanisms to compensate for missing facial information. Note that in our pilot behavioral studies, naïve observers categorized the normal schematic faces as faces, but not the rearranged schematics. These observations support the idea that FFA responses parallel the subjective experience of face perception (Hasson et al., 2001; Ishai et al., 2002; Grill-Spector et al., 2004). In our experiments, partial information of natural face parts or their correct configuration were each sufficient to activate the FFA well above the level of non-face objects. This responsiveness to incomplete face information resembles similar effects reported for object selective responses in the lateral occipital complex (Lerner et al., 2004) and may be a general property of the FFA when viewing face-like stimuli in the presence of contextual cues. The significance of these completion mechanisms in FFA's responsiveness to isolated facial information or contextual cues remains to be more systematically determined during face-identification tasks.

A limitation in our study was that we did not vary subjects' task during fMRI and only used face stimuli with a neutral expression. Future experiments that include a wider range of tasks and face stimuli are needed to determine the effect of 1st order configuration and part information during specialized processing of facial emotions, communicative expressions or identity by the AMG, STS, or the FFA respectively. Also, we focused our ROI analyses to only three brain regions as we were: (i) guided by the results of the group analysis in Experiment 1, (ii) motivated to test the hypothesis that FFA responses are particularly sensitive to prior experience with face and non-face stimuli, and (iii) limited in terms of statistical power for a more comprehensive analysis (due to the small sample size in Experiment 2). Future studies of additional brain regions, which are thought to be part of the core or extended face-processing network are needed for a more comprehensive view of how configural and part information in faces are represented across this network.

## Conclusion

Face perception is thought to involve the coordinated activity of a distributed neural system in humans that consists of multiple, face-selective regions including the AMG, STS, and FFA. It has been suggested that the AMG and STS represent changeable aspects of a face, extracting socially relevant meaning from faces, and the FFA mediates the visual analysis of faces representing their invariant aspects important in face detection and recognition. Our results show that during viewing of neutral faces, the STS and AMG responses are relatively invariant to removal of the natural details of the face as long as the typical face configuration is retained. In contrast FFA responses are invariant to either removal of the typical face configuration or the natural details of the face parts, but sensitive to simultaneous removal of both types of information. These findings emphasize the distinct representations of the typical face configuration and natural appearance of parts in the AMG and STS vs. FFA, demonstrating each region's sensitivity to different visual information in the face.

### Conflict of interest statement

The authors declare that the research was conducted in the absence of any commercial or financial relationships that could be construed as a potential conflict of interest.
