# Supplementary figures and images for: Distinct representations of configural and part information across multiple face-selective regions of the human brain
Source: Front Psychol. 2015 Nov 6;6:1710. doi: 10.3389/fpsyg.2015.01710 (PMC4635218; doi:10.3389/fpsyg.2015.01710)

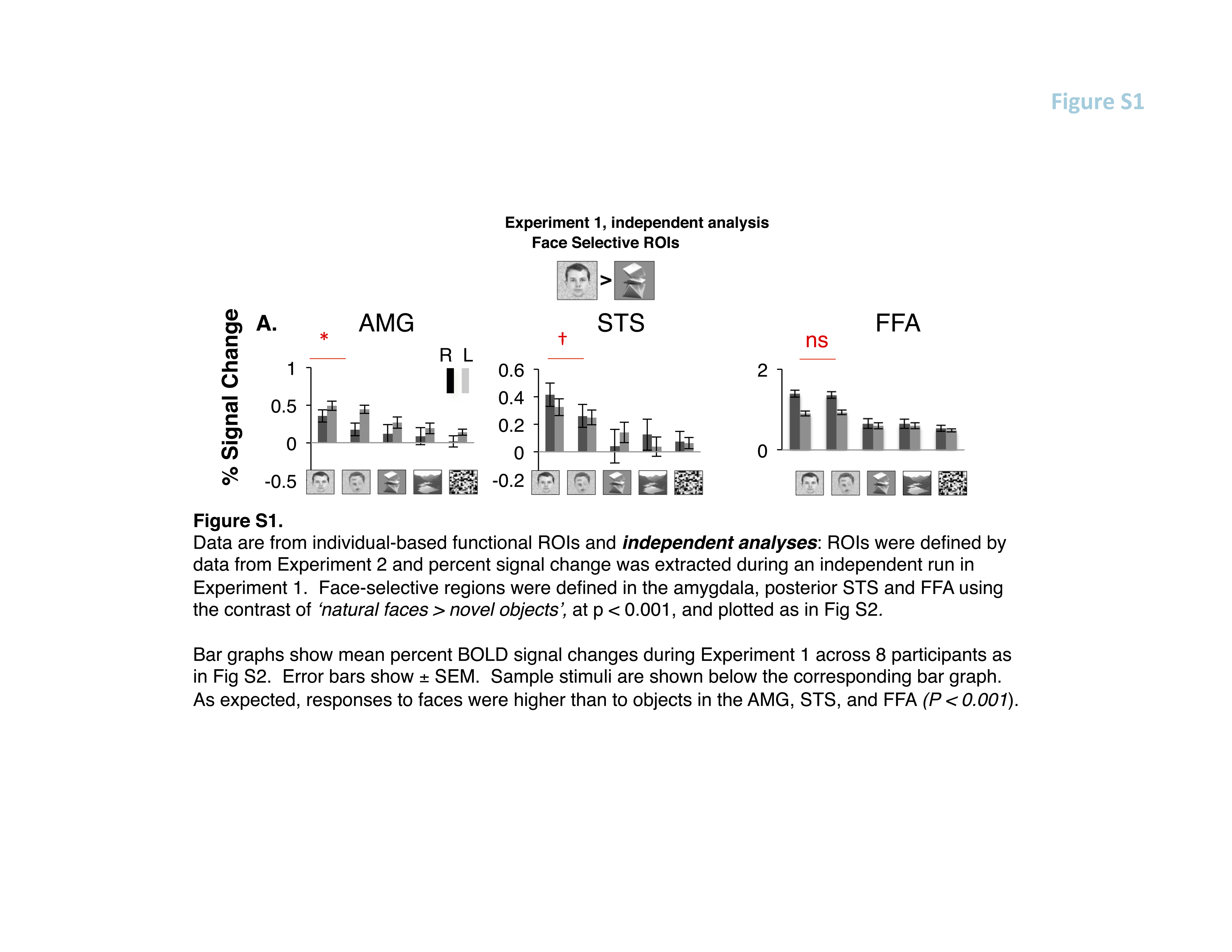

Supplement: Supplementary file 1 [file Image1.TIF]

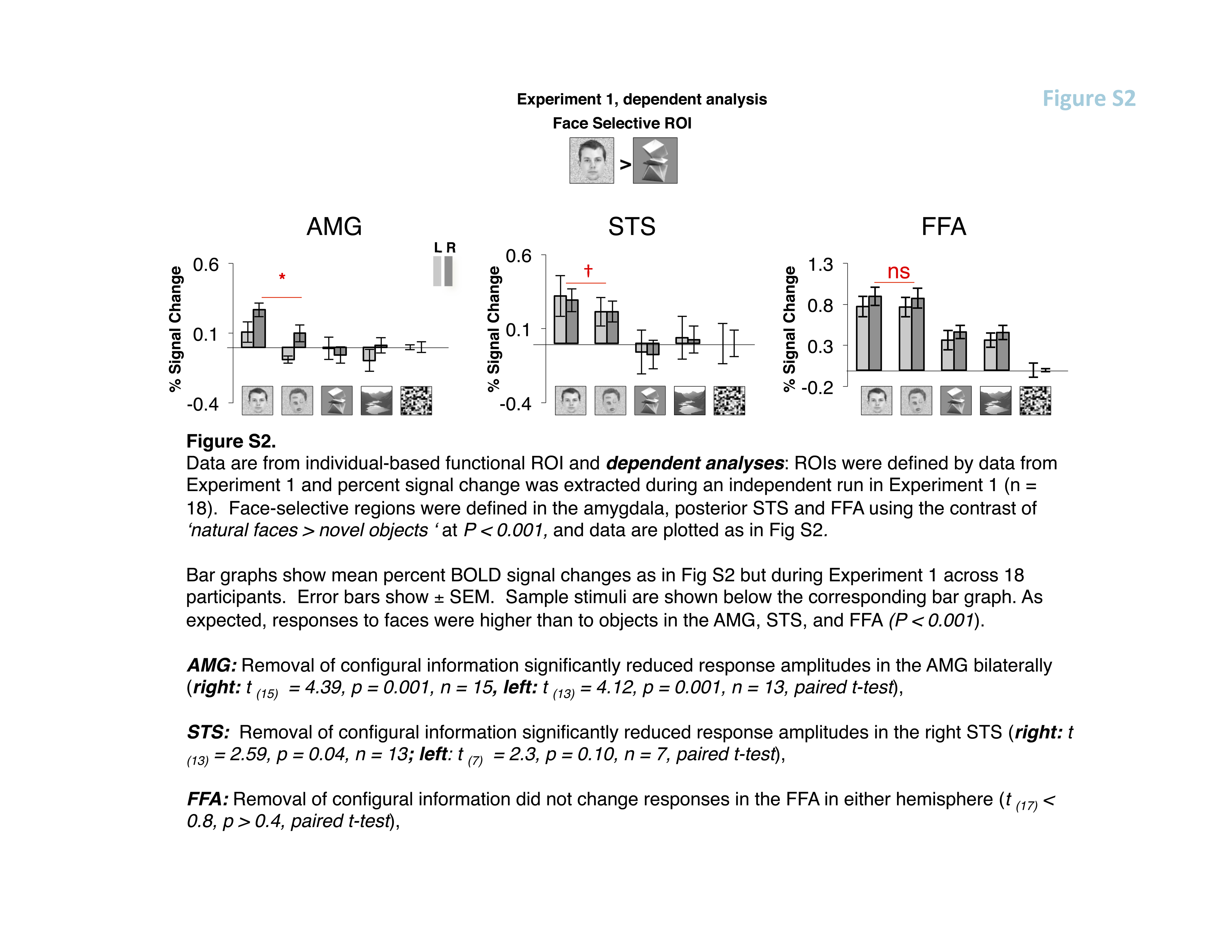

Supplement: Supplementary file 2 [file Image2.TIF]

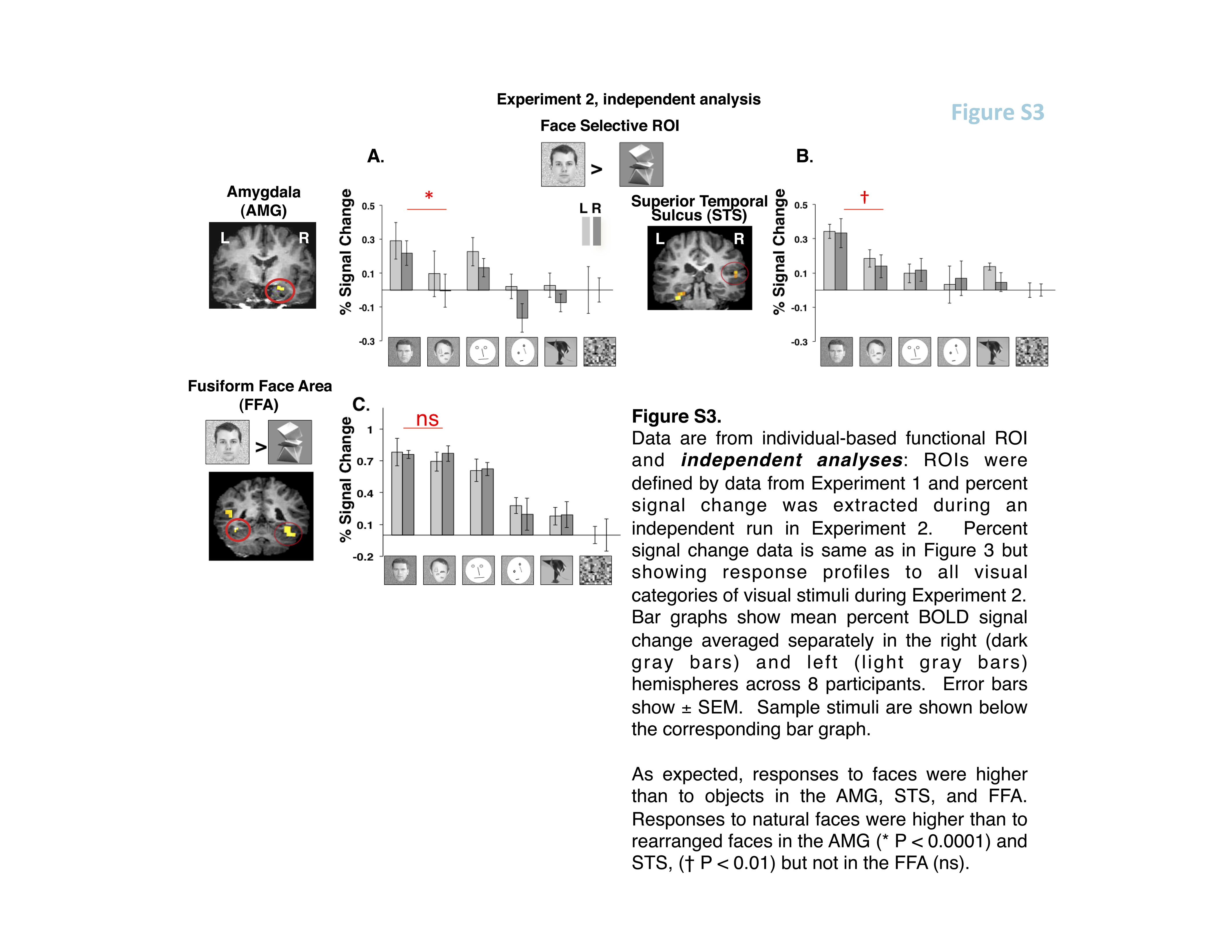

Supplement: Supplementary file 3 [file Image3.TIF]
