# Supplementary material for: Distinct representations of configural and part information across multiple face-selective regions of the human brain
Source: Front Psychol. 2015 Nov 6;6:1710. doi: 10.3389/fpsyg.2015.01710 (PMC4635218; doi:10.3389/fpsyg.2015.01710)
Supplement: Supplementary file 4 [file Table1.PDF]

Table S1. Regions showing BOLD signal increases in group-averaged data ($p < 0.001$, uncorrected, extent threshold of three voxels)

A. Natural faces > Rearranged natural faces

Anatomical Region	left/right	BA	x	y	z	Z
Brain Stem	R		6	-35	-7	5.06
Amygdala	R		22	-6	-10	4.40
Occipital-Lingual Gyrus	L	17	-12	-89	1	4.34
Caudate	R		26	-38	11	4.04
Superior Temporal Gyrus	R	22	57	-48	13	3.93

B. Rearranged natural faces > Natural faces

Anatomical Region	left/right	BA	x	y	z	Z
Middle Occipital Gyrus	R	19	36	-81	15	9.18
	L		-30	-82	21	9.01
Fusiform Gyrus	R	19	28	-66	-16	7.91
	R	37	26	-41	-11	6.07
	L	37	-34	-57	-11	4.68
Precuneus/Superior Parietal	R	7	24	-62	47	6.19
	L	7	-24	-62	49	6.28
	L	7	-22	-62	34	4.85
Inferior Parietal	R	40	36	-38	38	6.27
	L	40	-40	-35	37	6.43
Middle Frontal Gyrus	R	6	28	-8	43	5.27
	L	6	-24	6	46	4.69

BA: Brodmann Area; x, y, z : Talairach coordinates; Z: z-statistic.
